# Bioimpedance-Measurement-Based Non-Invasive Method for In Ovo Chicken Egg Sexing

**DOI:** 10.3390/bios13040440

**Published:** 2023-03-30

**Authors:** Congo Tak Shing Ching, Chien-Kai Wang, Pin-Chi Tang, Minh-Khue Ha, Chin Li, Hsuan-Ni Chiu, Fiona Yan-Dong Yao, Nguyen Chi Nhan, Nguyen Van Hieu, Thien-Luan Phan

**Affiliations:** 1 Graduate Institute of Biomedical Engineering, National Chung Hsing University, Taichung 402, Taiwan; tsching@dragon.nchu.edu.tw; 2 Department of Electrical Engineering, National Chi Nan University, Puli Township 54561, Taiwan; 3 Department of Animal Science, National Chung Hsing University, Taichung 402, Taiwan; 4 The iEGG and Animal Biotechnology Center, National Chung Hsing University, Taichung 402, Taiwan; 5 Department of Physics and Electronic Engineering, University of Science, Vietnam National University of Ho Chi Minh City, Ho Chi Minh City 700000, Vietnamncnhan@hcmus.edu.vn (N.C.N.); 6 Division of Science, Engineering and Health Studies, College of Professional and Continuing Education, The Hong Kong Polytechnic University, Hong Kong

**Keywords:** egg sexing, egg gender, non-invasive, in ovo, bioimpedance

## Abstract

Day-old male chick culling is one of the world’s most inhumane problems in the poultry industry. Every year, seven billion male chicks are slaughtered in laying-hen hatcheries due to their higher feed exchange rate, lower management than female chicks, and higher production costs. This study describes a novel non-invasive method for determining the gender of chicken eggs. During the incubation period of fourteen days, four electrodes were attached to each egg for data collection. On the last day of incubation, a standard polymerase chain reaction (PCR)-based chicken gender determination protocol was applied to the eggs to obtain the gender information. A relationship was built between the collected data and the egg’s gender, and it was discovered to have a reliable connection, indicating that the chicken egg gender can be determined by measuring the impedance data of the eggs on day 9 of incubation with the four electrodes set and using the self-normalization technique. This is a groundbreaking discovery, demonstrating that impedance spectroscopy can be used to sex chicken eggs before they hatch, relieving the poultry industry of such an ethical burden.

## 1. Introduction

The demand for hatching hens or roosters varies due to the different production purposes of the poultry industry. Males are only preferred as breeding objects in the production of meat and poultry strains due to their higher feed exchange rate, lower management, and higher production costs [1]. One of the most serious ethical issues in modern egg-producing poultry farms is the annual culling of billions of day-old male chicks. Male domesticated birds are not raised on poultry farms because they cannot lay eggs or put on enough flesh for meat production. It is estimated that 7 billion day-old male chicks from laying-hen hatcheries are killed globally each year [1,2].

Traditionally, newly hatched chicks can be identified for genital morphology at the age of one day [3,4] by trained and experienced chicken sexers, who can achieve a success rate of up to 95~98%, but training and accumulating experience takes two to six years, and there is a global shortage of sufficient human resources for this specific identification work [5]. Moreover, after gender identification, male and female chicks that are unsuitable for production require a euthanasia plan as well as chemical treatment equipment. These manpower allocations and processing work have become additional operating costs for the poultry industry, as well as a major ethical problem in terminating formed lives. Animal welfare issues are becoming increasingly important in both developed and developing countries. EU legislation, for example, has facilitated market segmentation by establishing trade standards for eggs (enriched cages, floor system, and free-range) and standardizing organic livestock production. Higher animal welfare standards may be implemented by industries in order to develop a national profile. Danish and German egg producers, for example, have agreed to stop trimming the beaks of layers [6]. The EU has also discussed whether law enforcement should be used to prevent the poultry industries from pursuing the gender elimination strategy in the future. However, in the absence of a fully developed technology to determine the sex of a chick before its life has begun, the law has been unable to prevent industries from continuing to do what they have been doing for decades.

A study from the University of Leipzig [7] published at the beginning of 2018 stated that fluorescence and Raman spectroscopy of blood offers the potential for precise and contact-free in ovo sex determination of chicken eggs during the fourth incubation day. Although this is a very fast and precise method, with only 3.5 days of incubation process, and the accuracy is said to be above 90% [7], such a type of spectroscopy requires a window on the eggshell, meaning this is an invasive method that affects the embryo, which leads to a decrease in hatching rates.

Many more studies have been conducted using the spectroscopic method, including German Agri Advance Technologies, French Tronico (financed by the government in 2017), and Canadian Hypereye (initiated in 2007, repaid by the government in 2018) [8,9,10]. There have been no results recorded, despite the fact that the majority of them pledged to deliver the technology between 2019 and 2020. This is yet more reason to conclude that this procedure is a long shot and that new approaches should be developed.

It was not until the late end of 2018 that the first fertilized egg’s gender detection technology was introduced to the commercial production of laying hens: the Seleggt method. This technology is derived from another detection technology also developed by the University of Leipzig, Germany. On the blunt end, the eggshells were cut open using a laser after 8 to 10 days, the estimated time when the hormone can be found in the egg [11,12,13,14], of incubating, then a small amount of egg content solution was extracted and used to measure the estrogen content. Since the amount of estrone sulfate in the female embryos is significantly higher than that in males at the time the hormone has presented in the egg, it can be used as a basis for identification [11,12,13,14]. Although compared to the manual identification methods this method is considered to have the same identification rate, due to the eggshell perforation sampling work, higher sanitary conditions are required for subsequent incubation facilities and environments to avoid possible pollution and incubation losses. Moreover, this commercial technology is currently using radioimmunoassay for the determination of estrin, so the relative technical cost and analysis time are not easy to reduce. In other words, the current commercialized technology still needs to complete the gender identification process through intrusive sampling and biochemical analysis. The direction of future technology development is to replace intrusion sampling with non-invasive methods and fast real-time analysis; photoelectric analysis equipment is an example.

Impedance spectroscopy is a well-established method for a variety of applications, including bacterial detection, biomarker detection, and microorganism viability determination [14,15,16,17,18,19]. The principle of this technique is to inject a constant current into the object to be measured and calculate its voltage to find out the electrical impedance response of the object. Based on a study by Burke and Sharp in 1989 [13], compared to hens, the eggs that hatched into roosters have a larger yolk, as well as a heavier fetus. Hence, it is logical to expect these eggs to have a different electrical impedance value. Because the eggshell can be thought of as the cell membrane, the protein as the cytoplasm, and the egg yolk as the nucleus, the theory of impedance spectroscopy can be logically applied in this circumstance.

BIA, or bioelectrical impedance analysis, is a widely used technique for estimating total body water and calculating fat and muscle mass based on total weight. Various BIA techniques can assess different components of body composition such as hydration status, muscle mass, and percentage of body fat. Body composition as measured by BIA has been associated with other nutritional measures, and mortality. The utilization of different electrode positions for body measurement has made it possible to obtain varied body composition measurements. Applying the BIA technique, we could investigate the variance in BIA data between male and female eggs. This work presents the development process of a method for non-invasive in ovo chicken egg gender identification applying the impedance spectroscopy technique. The gender is then confirmed after the incubation process by using the standard PCR chicken egg gender identification process. The presented work involves an integrated system of measuring electrodes and an incubation machine, which will be a great resource for the future development of a commercially available technology in non-invasive chicken egg sexing in the poultry industry, solving a huge ethical problem globally, where billions of one-day-old male chicks are culled annually.

## 2. Materials and Methods

### 2.1. Eggs and Chemical Preparation

A total of 17 fertilized chicken eggs (Kinmen county, Taiwan breed) were obtained from the Department of Animal Science, National Chung Hsing University.

Potassium permanganate (KMnO4 = 158.04) and formalin (HCHO = 30.02) extra pure reagent was purchased from Choneye Pure Chemical (Taipei, Taiwan). Blood Genomic DNA purification kit for PCR gender identification was purchased from Biokit (Barcelona, Spain).

### 2.2. Chicken Egg Electrodes’ Positions

**Figure 1:**
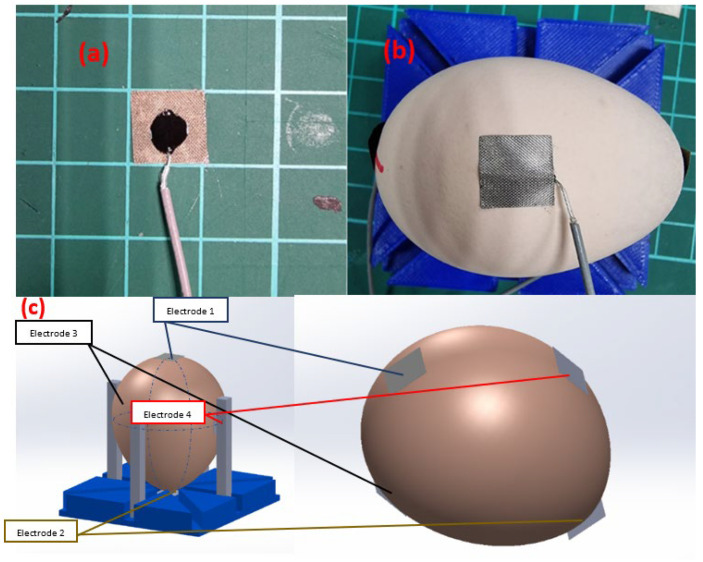
(**a**) Carbon epoxy was dropped onto 10 × 10 (mm^2^) adhesive conductive cloth (**b**) taped onto designated points on a chicken egg using RG-1.13 mm coaxial cable on the (**c**) designated electrode position.

In this study, a 3D printed simple device consisting of four movable columns (see Figure 1c) was utilized to mark the circumferences of the eggs, along with the positions of the electrodes. These positions were based on the established bioelectrical impedance analysis standards for body mass assessment. Specifically, two electrodes were affixed to the broad and narrow ends of each egg, corresponding to position 1 and position 2 (determined by the height circumference). Position 3 and position 4 were identified by marking the width circumference of the egg, with position 3 being an arbitrary point on this circumference and position 4 located diametrically opposite to position 3. Notably, the positions of the electrodes were consistent across all eggs in the study, and after marking the positions, the 3D printed device was no long used for the next step.

### 2.3. Carbon-Epoxy-Based Electrode

The performance of the electrode is crucial to the impedance spectroscopy approach. The electrode needs to be accurate, repeatable, and low noise. An ordinary ECG electrode was utilized for the development process’ evaluation phase.

Approximately 40 μL of carbon epoxy (volume resistivity of ≈40 Ohms∙cm) was dropped onto a 10 × 10 (mm^2^) adhesive conductive cloth (contact resistance of 0.05 Ohms/cm^2^) (Figure 1a) where the cloth was used to stick on one end of the RG-1.13 mm coaxial cable to the designated point on the chicken egg (Figure 1b,c). Using the resistivity (ρ) equation

ρ=R∗A/l

where *R* is the resistance, *A* is the cross-sectional area, and *l* is the length of the specimen, plus the resistance of the 1 cm^2^ conductive cloth piece, the total resistance of the electrode was calculated to be 2.05 Ohm.

### 2.4. Pinout Circuit

**Figure 2:**
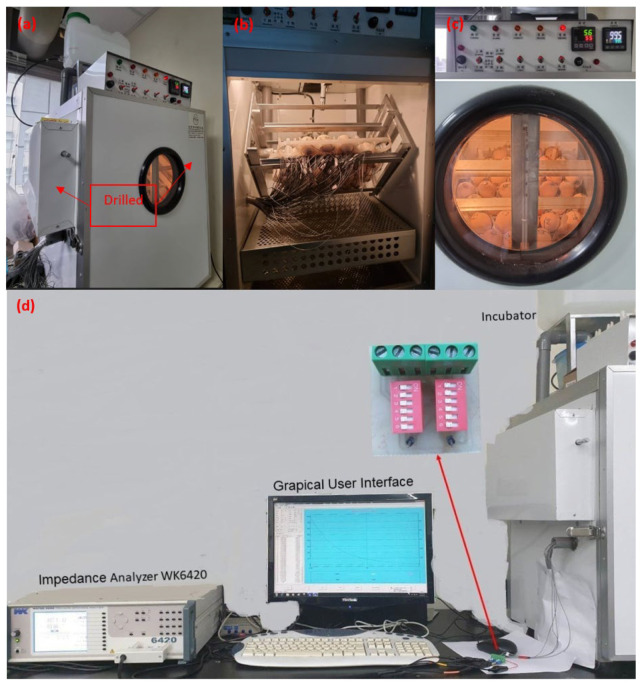
(**a**,**b**) A small 20 mm diameter hole was drilled into the incubator wall in order to enable the cables to be tunneled out. (**c**) The machine settings were set to maintain a consistent humidity level of 55%, and a temperature of 37.5 °C. (**d**) The cables were then connected to a pinout circuit, and the impedance data were measured using a precision impedance analyzer.

The eggs were connected to one end of the coaxial cables, while the other end was linked to a pinout circuit (Figure 2d) with headers connecting to the egg electrodes. The pinout circuits serve two main purposes: simplifying the process of switching between electrode combinations during measurements and providing shielding for the cables. To ground the pinout circuit, the mesh of the coaxial cables was stripped open and connected to one of the headers. This allowed the ground of the impedance analyzer (black crocodile clip, Figure 2d) to be connected to the ground of the pinout circuit. Finally, the probes of the impedance analyzer were attached to the pins in order to conduct the measurement.

### 2.5. Experimental Setup

During the 14-day incubation process from day 0 to day 13, the eggs obtained were kept within the confines of an incubator. To facilitate measurements while the eggs were still within the incubator, the signal wires were tunneled out (refer to Figure 2a,b), and the impedance analyzer probes were connected to the pinout circuits. This setup enabled the collection of measurements while the eggs remained in the incubator.

#### 2.5.1. Incubator Preparation

In order to maintain appropriate levels of humidity during the incubation process, deionized water was utilized as the medium. The humidity was maintained at a stable range of 54–56%, while the temperature was kept constant at 37.5 °C (as depicted in Figure 2c) [20,21,22]. Prior to the commencement of each incubation process, it was necessary to sterilize the incubation area. This was accomplished through the customary fumigation procedure utilizing formalin and potassium permanganate.

#### 2.5.2. Impedance Measurement Experiment

The bioimpedance data were measured using the Wayne Kerr 6420C Impedance Analyzer & LCR Meter (Wayne Kerr Electronics, Massachusetts, USA) as shown in Figure 2. The specified parameters for measurement were 100 mV rms voltage, with measuring frequency range of 20 Hz to 10 MHz (100 points), and measurement speeds of 80 ms per measurement.

On the day of obtaining the eggs, electrode attachments were made at a temperature of approximately 20 degrees Celsius. The carbon-epoxy-based electrodes were allowed to cure at room temperature for 24 h before placing the eggs inside the incubator. A small hole with a diameter of 20 mm was drilled and used for tunneling the cables out as depicted in Figure 2a,b. To ensure a sealed environment, a 20 mm PVC tube was inserted into the hole and kept closed throughout the incubation period.

The impedance measurement experiment was carried out in the following steps:

a. A dry, clean towel was used to clean the egg’s surface before it was numbered. The temperature of the egg storage area was maintained at around 20 °C.
b. Conductive fabric cloth was cut into pieces that were 10 mm × 10 mm in size.
c. Coaxial cables that were about 1 m long were cut, striped, and had the shield layer insulated with heat-shrunk tubing.
d. Forty cubic millimeters of carbon epoxy was dropped onto the conductive fabric cloth.
e. The coaxial cable’s stripped end was inserted into the carbon-epoxy-dropped conductive fabric cloth.
f. The entire arrangement was taped to the desired positions of each egg.
g. They were allowed to cure for 24 h in a 20 °C climate-controlled room.
h. The eggs were placed inside the incubator, the wires tunneled out and connected to SMA heads and pinout circuit.
i. Every other 24 h, an impedance spectrum measurement was made for 13 more days.

#### 2.5.3. Genomic DNA Extraction and Gender Identification Using PCR

**Figure 3:**
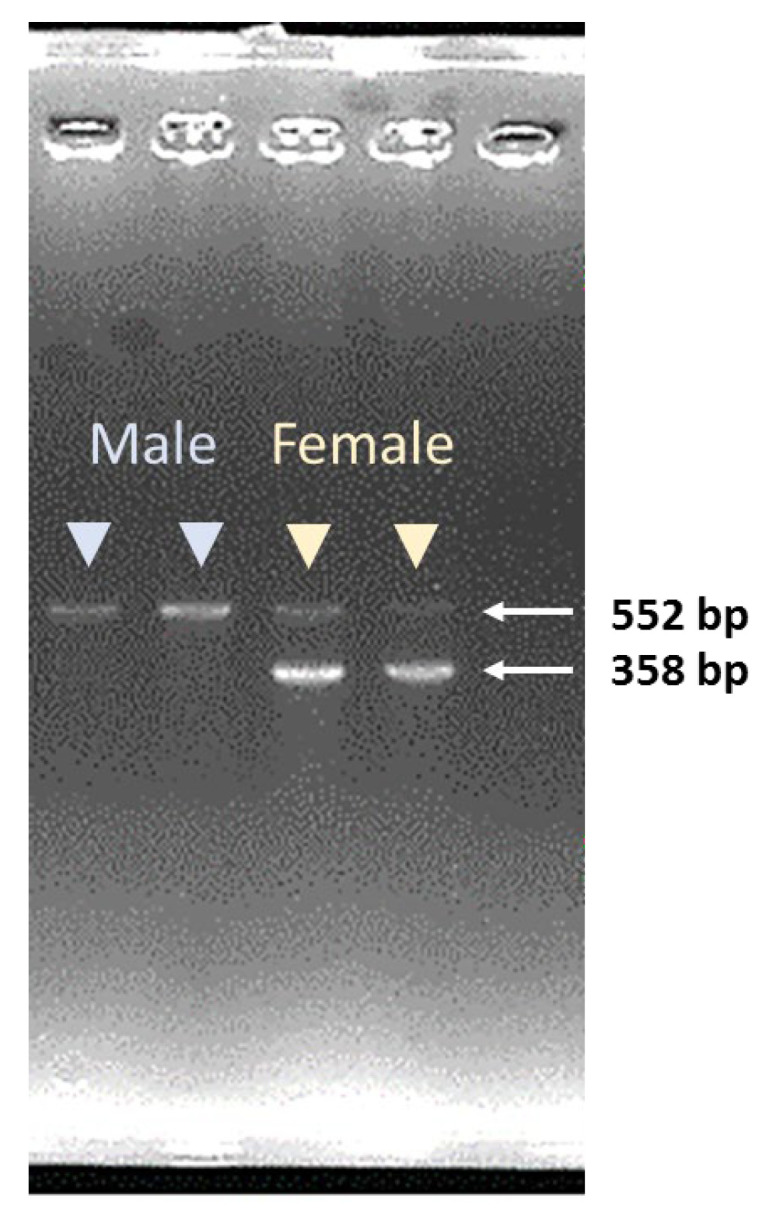
Sex identification for chick fetus using PCR. One single band at 552 bp indicated a male fetus and two bands at 552 bp and 358 bp indicated a female fetus.

A standard PCR process, as described in [23], was used to determine the gender of the chicken egg after day 13 of incubation. Genomic DNA was collected from the chick’s blood and applied to the PCR. PCR was performed as Table 1. with each sample containing a total 25 µL mixture of 2 µL eluted genomic DNA, 16 µL deionized water, 5 µL master mix, 1 µL 2250 forward primer (5′-GTTACTGATTCGTCTACGAGA-3′), and 1 µL 2718 reverse primer (5′-ATTGAAATGATCCAGTGCTTG-3′) using Biometra T-Personal thermal cycler (Analytik Jena GmBH, Jena, Germany). The primer design is based on a pilot study for gender identification on avians (Romanova et al., 2019). Agarose gel (2%) electrophoresis was performed to analyze PCR products. Single bands of 552 bp indicated a male fetus, whereas double bands in 358 bp and 552 bp indicated a female fetus (Figure 3).

### 2.6. Statistical Analysis

The concept of self-normalization, as introduced in Phan et al. (2021) [15], was utilized to investigate the parameter affected by noise in the spectrum and to minimize the impact of variations between different embryos during data analysis.

Independent *t*-test statistical analysis using Microsoft Excel were made in order to construct the relationship between the chicken egg sex and the collected impedance data. An independent *t*-test was made for each and every day of the incubation process, for all of the measured spectra.

### 2.7. Experimental and Analyzing Flow

**Figure 4:**
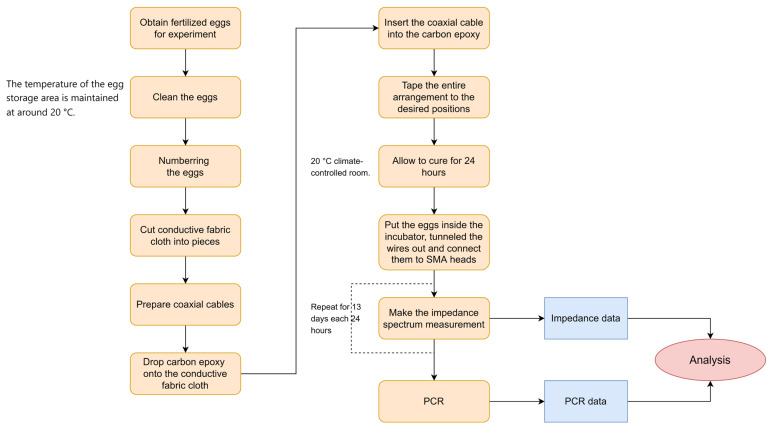
Experimental flowchart for impedance measurement and data analysis.

A full flowchart of the methodology is included in Figure 4.

## 3. Results and Discussion

### 3.1. Electrode Evaluation Test

Two different tests were used to validate the electrode performance. To reduce electrode impedance error, the first step is to measure the electrodes’ resistance and ensure that it is constant. Unfertilized eggs are used in the second test. The performance of the electrode can be verified by observing its impedance spectrum over the course of eleven days both inside and outside of the enclosure.

**Figure 5:**
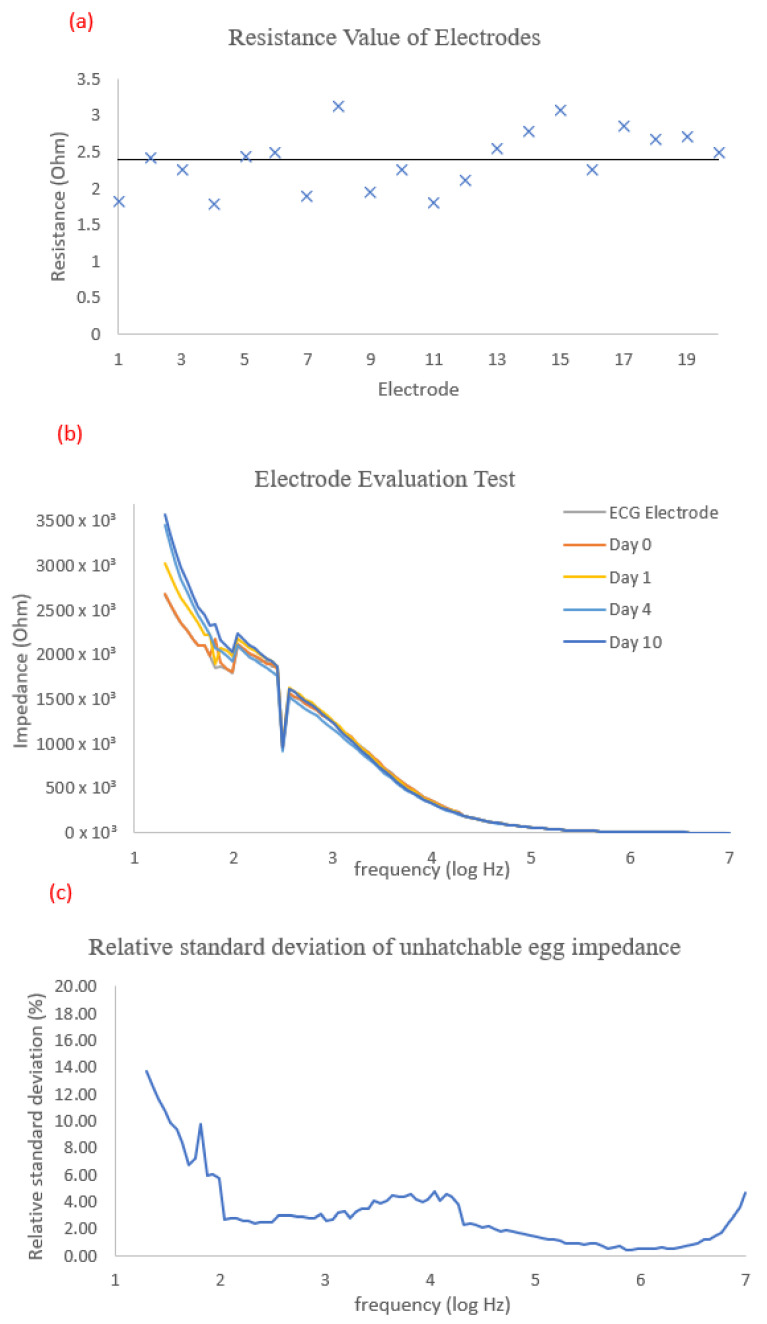
(**a**) Electrodes’ resistance validation (each sign represent the value of one electrode). (**b**) Impedance data of an unfertilized egg at different days with (**c**) relative standard deviation.

Without taping onto the egg, a total of 20 electrode configurations were prepared. They were tested for resistance using a Fluke 179 true-rms multimeter. The measured resistance was discovered to range from 1.78 to 3.12 with an average of 2.383, as shown in Figure 5a. This was found to be in agreement with the electrodes’ calculated resistance value from the fabrication of the carbon epoxy electrodes above.

A pair of ECG electrodes were attached to the blunt and pointy ends of an unfertilized egg to measure its impedance. After the measurement, the ECG electrodes were removed, and a pair of designed electrodes were attached to positions 1 and 2 on the egg surface that had been cleaned with a dry towel. The impedance spectrum was then measured after 24 h at room temperature. The egg was then placed back in the incubator for an hour at a temperature of 37.5 degrees Celsius, and Day 0 of the incubation measurement was recorded. The impedance was measured again on Days 1, 4, and 10 of the incubation process to assess electrode performance in an environment with a constant humidity of 55% and temperature of 37.5 °C. The impedance value varied at low frequencies (20 Hz to 100 Hz), but the agreement level was acceptable from 100 Hz and above with less than 5% error. Figure 5b illustrates the impedance spectrum.

### 3.2. Chicken Egg Gender Evaluation Test

#### 3.2.1. PCR Chicken Egg Sex Result

Seventeen fertilized eggs from Kinmen county, obtained from the Department of Animal Science at National Chung Hsing University in Taichung, Taiwan, were analyzed using PCR for chick egg sex determination. Of these, one egg was unfertilized, while eight were male and eight were female. A summary of the results is presented in Table 2.

#### 3.2.2. Impedance Spectrum Result Data

**Figure 6:**
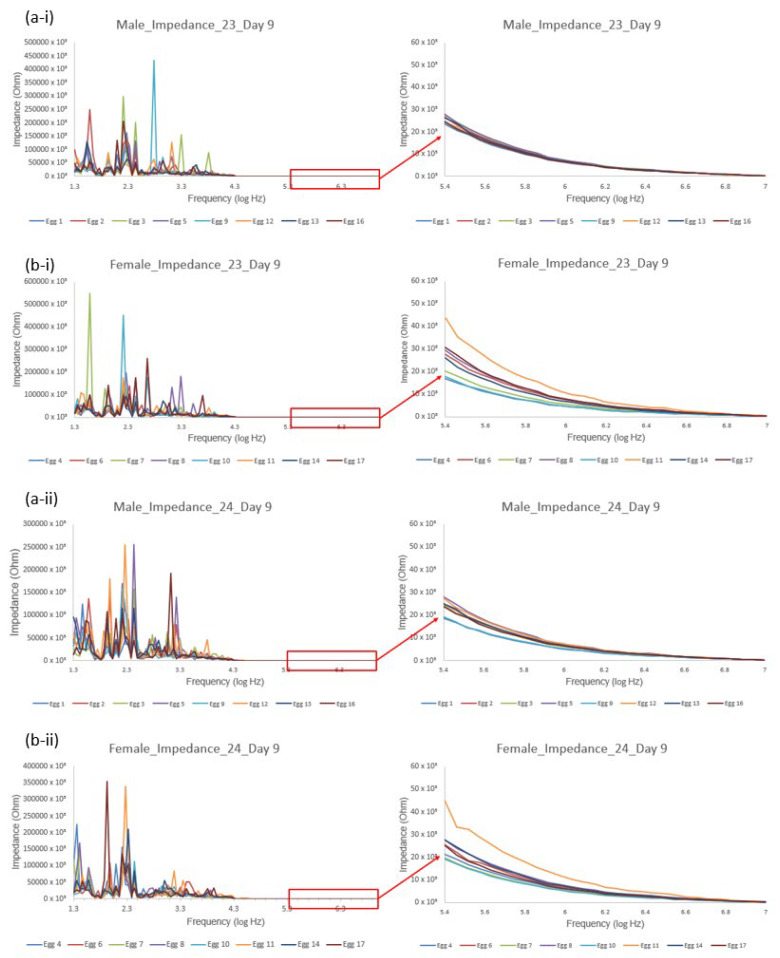
Impedance spectrum measurement of male (**a**) and female (**b**) eggs between electrodes at position 2–3 (**i**) and 2–4 (**ii**) on Day 9.

Figure 6 presents the impedance data from eight male and eight female eggs on day 9 of incubation, with electrodes positioned at 2–3 and 2–4. Despite the presence of noise, repeating peaks can be observed at various frequencies in the impedance spectrum. As an unfertilized egg was utilized to test the electrode and readout circuit, it can be safely assumed that the collected impedance data were solely that of the egg and the internal variations that occurred, including the embryo’s development and protein adjustments in the eggshell. At frequencies of 200 kHz and higher, the spectrum exhibits less noise, potentially due to high-frequency signals penetrating the eggshell. Given that the egg can be viewed as a cell structure, with the eggshell serving as the cell membrane, higher frequencies are more favorable for passing through this capacitor and transmitting the signal into and out of the eggshell. This cell membrane is often represented by capacitors as an equivalent circuit.

**Figure 7:**
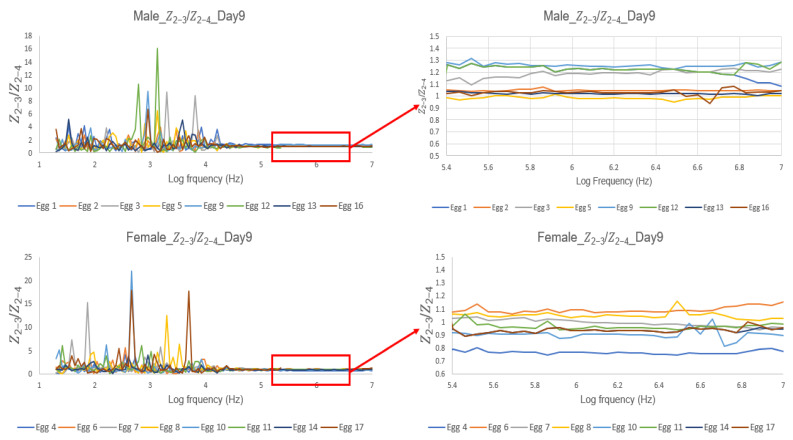
The self-normalized spectrum of impedance value of pair 2–3 divided by impedance value of pair 2–4.

The use of ratio data enabled a more impartial and reliable identification of gender differences, as demonstrated in Figure 7. Notably, characteristic curves were observed at higher frequencies, despite significant variations in the low frequency range (20 Hz to 100 kHz). At the frequency range of 300 kHz to 10 MHz, the impedance ratio of both male and female eggs remained stable. Mean values for male and female eggs were calculated and plotted against the days of data collection at different frequencies to observe any changes over time. This allowed for the determination of the frequency at which the egg’s gender can be identified by comparing the ratio values between electrode positions 2–3 and 2–4.

It is clear from the spectrum that the ratio data for male eggs in the frequency range of 300 kHz to 10 MHz has a value greater than 1, whereas the ratio value for female eggs almost always falls below 1. However, statistical techniques must be used to demonstrate that the difference can be used to accurately determine the gender of the egg.

The ratio of impedance measurements between positions 2–3 and 2–4, as determined by a statistically independent t-test, significantly correlates with the gender of chicken eggs. The ideal measurement range was found to be throughout day 9 until the end of the incubation process on day 13 at 376 kHz up to 2.79 MHz.

In the ideal frequency range, the measurement of male and female eggs revealed significant differences, with the lowest *p*-value of the independent *t*-test occurring at the frequency of 376 kHz. The impedance ratio values of male and female eggs always demonstrated some degree of distinction from the start of the incubation process, according to Table 3. The significant difference discovered at this range on day 9 persisted through the remaining incubation days, until day 13, when the incubation process came to an end.

Burke and Sharp’s study [13] demonstrated a high level of expertise in the subject matter. According to their research, there is a difference in the weight of male and female embryos during the early stages of incubation, but this difference only becomes significant after the 11th day. In the presented experiment, a technique similar to bioimpedance analysis was utilized and was able to non-invasively reach a similar conclusion. A significant difference between the genders of the eggs was found, in ovo, on the 9th day of incubation, as supported by a consistent *p*-value of less than 0.05.

**Table 3:** Mean impedance ratio value of male and female egg throughout the 13 days of incubation measured at the frequency of 376 kHz.

Incubation Day	Male		Female		*p*-Value of Independent Two Tail *t*-Test
	Mean	SD	Mean	SD	
1	1.125607	0.167139	0.95094	0.086574	0.023633
2	1.023389	0.16627	0.95714	0.072835	0.326269
3	1.06362	0.123114	0.974016	0.177845	0.264056
4	1.087815	0.12767	0.943842	0.070731	0.017585
5	1.061307	0.119568	1.006839	0.154494	0.444491
6	1.117094	0.172387	0.959187	0.101615	0.047364
7	1.058761	0.148037	0.963327	0.115739	0.174494
8	1.066774	0.125547	0.990487	0.119059	0.232832
9	1.122262	0.112674	0.953107	0.124892	0.006125 *
10	1.083995	0.128271	0.928134	0.07559	0.012954 *
11	1.077437	0.120659	0.94658	0.076244	0.023521 *
12	1.131611	0.167438	0.961139	0.102673	0.030321 *
13	1.08715	0.095502	0.959394	0.091049	0.016 *

Remark: * shows a continuous *p*-value of <0.05.

## 4. Conclusions

A pioneer study was conducted on a non-invasive method for chicken egg gender identification. The result indicates that this is a promising humane method for mass egg production poultry industries. The gender of the eggs can be determined by comparing the impedance of fertilized chicken eggs on day 9 of incubation at 376 kHz between electrode positions 2–3 and 2–4. Future research on electrode optimization should focus on electrode material, electrode impedance stabilization, and electrode positions. Additionally, a larger sample pool will be taken into account to boost the method’s precision.

## Figures and Tables

**Table 1:** PCR program for chicken gender identification.

	Temperature	Time	
Initial	95 °C	30 s	
Denaturation	95 °C	30 s	30 cycle
Annealing	58 °C	1 min	
Extension	72 °C	1 min	
Finish	72 °C	5 min	

**Table 2:** Survival and gender records for 14 days incubating of fetal Kinmen county chicken.

No.	Survival Status		Gender
1	+	Good	M
2	+	Good	M
3	+	Good	M
4	+	Good	F
5	+	Good	M
6	+	Good	F
7	+	Good	F
8	+	Good	F
9	+	Good	M
10	+	Good	F
11	+	Good	F
12	+	Good	M
13	+	Good	M
14	+	Good	F
15	N/A	N/A	N/A
16	+	Good	M
17	+	Good	F

Remarks: N/A: not fertilized, +: fertilized, M: male, F: female.

## Data Availability

Raw data were generated at the Graduate Institute of Biomedical Engineering, College of Engineer, National Chung Hsing University, Taichung, Taiwan. Derived data supporting the findings of this study are available from the corresponding authors Congo Tak Shing Ching and Thien-Luan Phan on request.
